# Supplementary material for: Heat adaptation of phage T7 under an extended genetic code
Source: Virus Evol. 2021 Dec 1;7(2):veab100. doi: 10.1093/ve/veab100 (PMC8923235; doi:10.1093/ve/veab100)
Supplement: veab100_Supp [file veab100_supp.zip › Sequencing_Analysis.zip!/Sequencing_Analysis/A2/evidence/JC_SIDE_1_43.html]

BRESEQ :: Evidence


| New junction evidence | | | | | | | | | | | |
| --- | --- | --- | --- | --- | --- | --- | --- | --- | --- | --- | --- |
|  | | seq id | position | reads (cov) | reads (cov) | score | skew | freq | annotation | gene | product |
| \* | ? | NC\_001604.1 | = 1252 | 0 (0.000) | 3 (0.010) | 3/580 | 23.4 | 100% | intergenic (–/–) | *–/–* | –/– |
|  | ? | NC\_001604.1 | 2742 = | 0 (0.000) |  |  |  |  | intergenic (–/–) | *–/–* | –/– |
| Rejected: Coverage evenness skew score above cutoff. | | | | | | | | | | | |

Only 100 of 478 total aligned reads displayed.  

`CGTTTTCGACCACGCTTACGAAATGCTGAAAGAAAACATCCGTTATGATGACATCCGTGACACTGATGACCTGCACGATGCTATTCACATGGCTGCCGATAATGCAGTTCCGCACTACTACGCTGACATCTTTAGCGTAATGGCAAGTGAGGGCATTGACCTTGAGTTCGAAGACTCTGGTCTGATGCCTGACACCAAGGACGTAATCCGCATCCTGCAAGCGCGTATCTATGAGCAATTAACGATTGACCTCTGGGAAGACGCAGAAGACTTGCTCAATGAATACTTGGAGGAAGTCGAGGAGTACGAGGAGGATGAAGAGTAATGTCTACTACCAACGTGCAATACGGTCTGACCGCTCAAACTGTACTTTTCTATAGCGACATGGTGCGCTGTGGCTTTAACTGGTCACTCGCAATGGCACAGCTCAAAGAACTGTACGAAAACAACAAGGCAATAGCTTTAGAATCTGCTGAGTGATAGACTCAAGGTCGCTCCTAGCGAGTGGCCTTTATGATTATCACTTTACTTATGAGGGAGTAATGTATATGCTTACTATCGGTCTACTCACCGCTCTAGGTCTAGCTGTAGGTGCA  >  NC_001604.1/954‑1549`  

                                                                                                                                                                                                                                                                                                          `|`                                                                                                                                                                                                                                                                                                           
 `ggtcatcagtgtcacggatgtcatcataacgaaaacatccgttctcatgacatccgtgacactgATGACCTGCACGATGCTATTCACATGGCTGCCGATAATGCTGTTCCACACTACTACGCTGACATCTTTCGCGTAATGGCAAGTGAGGGCATTGACCTTGAGTTCGAAGACTCTGTTCTGATGCCTGACACCAAGGACGTAATCCGCATCCTGCCTGCGCGTATCTATGAGCAATTAATGATTGACCTCTTGGAAGACTCAGAAGACTTCCTCAATGTCTCCTTGGAGGAAGTCGAGG                                                                                                                                                                                                                                                                                                        >  1:177500‑M1/31‑301 (MQ=255)`  
       `cacCACGCTTACGAAATGCTGAAACAAAACATCCGTTATGATGACATCCGTGACACTGATGACCTGCACGATGCTATTCACATGGCTGCCGATAATGCAGTTCCGCACTACTACGCTGACATCTTTAGCGTAATGGCAAGTGAGGGCATTGACCTTGAGTTCGAAGACTCTGGTCTGATGCCTGACACCAAGGACGTAATCCGCATCCTGCAAGCGCGTATCTATGAGCAATTAACGATTGACCTCTGGGAAGACGCAGAAGACTTGCTCAATGAATACTTGGAGGAAGTCGAGGAagtcg                                                                                                                                                                                                                                                                                                  <  1:41677‑M1/300‑6 (MQ=255)`  
            `cGCTTACGCAATGCTGAAAGAAAACATCCGTTATGATGACATCCGTGACACTGATGACCTGCACGATGCTATTCACATGGCTGCCGATAATGCAGTTCCGCACTACTACGCTGACATCTTTAGCGTAATGGCAAGTGAGGGCATTGACCTTGAGTTCGAAGACTCTGGTCTGATGCCTGACACCAAGGACGTAATCCGCATCCTGCAAGCGCGTATCTATGAGCAATTAACGATTGACCTCTTGGAAGACGCAGAAGACTTGCTCAATGAATACTTGGAGGAAGTCGcacgctgtctcttt                                                                                                                                                                                                                                                                                             >  1:398466‑M1/1‑287 (MQ=255)`  
                           `gaaagaaaACATCCGTTATGATGACATACGTGACACTGATGACCTGCACGATGCTATTCACATGGCTGCCGATAATGCAGTTCCGCACTACTACGCTGACATCTTTAGCGTAATGGCAAGTGAGGGCATTGACCTTGAGTTCGAAGACTCTGGTCTGATGCCTGACACCAAGGACGTAATCCGCATCATGCAAGCGCGTATCTATGAGCAATTAACGATTGACCTCTGGGAAGACGCAGAAGACTTGCTCAATGAATACTTGGAGGAAGTAGAGGAagtcgcacgacagaaagaaattgac                                                                                                                                                                                                                                                                              >  1:86414‑M1/1‑276 (MQ=255)`  
                                      `cccgTTATGATGACATCCGTGACACTGATGACCTGCACGATGCTATTCACATGGCTGCCGATAATGCAGTTCCGCACTACTACGCTGACATCTTTAGCGTAATGGCAAGTGAGGGCATTGACCTTGAGTTCGAAGACTCTGGTCTGATGCCTGACACCAAGGACGTAATCCGCATCCTGCAAGCGCGTATCTATGAGCAATTAACGATTGACCTCTGGGAAGACGCAGAAGACTTGCTCAATGAATACTTGGAGGAAGTCGAGGAagtcgcacgacagaaagaaattgaccgcgctaaggc                                                                                                                                                                                                                                                                   <  1:507863‑M1/300‑37 (MQ=255)`  
                                              `gataaCATCCGTGACACTGATGACCTGCACGATGCTATTCACATGGCTGCCGATAATGCAGTTCCGCACTACTACGCTGACATCTTTAGCGTAATGGCAAGTGAGGGCATTGACCTTGAGTTCGAAGACTCTGGTCTGATGCCTGACACCAAGGACGTAATCCGCATCCTGCAAGCGCGTATCTATGAGCAATTAACGATTGACCTCTGGGAAGACGCAGAAGACTTGCTCAATGAATACTTGGAGGAAGTCGAGGAagtcgcacgacagaaagaaattgaccgcgctaaggcccgtaaag                                                                                                                                                                                                                                                           >  1:619489‑M1/1‑257 (MQ=255)`  
                                                 `cgtgggctcggagatgtgtataagagacaggctattcacatggctgccgataatgcagttcCGCACTACTACGCTGACATCTTTAGTGTAATGGCAAGTGAGGGCATTGACCTTGAGTTCGAAGACTCTGGTCTGATGCCTGACACCAAGGACGTAATCCGCATCCTGCAAGCGCGTATCTATGAGCAATTAACGATTGACCTCTGGGAAGACGCAGAAGACTTGCTCAATGAATACTTG‑‑‑‑‑‑‑‑‑GAGGAagtcgcacgacagaaagaaattgaccgcgctaaggcccgtaaagaacgtcacgagg                                                                                                                                                                                                                                               <  1:446741‑M1/271‑57 (MQ=255)`  
                                                    `aTCCGTGACACTGATGACCTGCACGATGCTATTCACATGGCTGCCGATAATGCAGTTCCGCACTACTACGCTGACATCTTTAGCGTAATGGCAAGTGAGGGCATTGACCTTGAGTTCGAAGACTCTGGTCTGATGCCTGACACCAAGGACGTAATCCGCATCCTGCAAGCGCGTATCTATGAGCAATTAACGATTGACCTCTGGGAAGACGCAGAAGACTTGCTCAATGAATACTTGGAGGAAGTCGAGGAagtcgcacgacagaaagaaattgaccgcgctaaggcacgtaaagaacgtc                                                                                                                                                                                                                                                     >  1:90316‑M1/1‑251 (MQ=255)`  
                                                      `cagggacacTGATGACCTGCACGATGCGATTCACATGGCTGCCGATAATGCAGTTCCGCACTACTACGCTGACATCTTTAGCGTAATGGCAAGTGAGGGCATTGACCTTGAGTTCGAAGACTCTGGTCTGATGCCTGACACCAAGGACGTAATCCGCATCCTGCAAGCGCGTATCTATGAGCAATTAACGATTTACCTCTGGGAAGACGCAGAAGACTTGCTCAATGAATACTT‑‑‑‑‑‑‑‑T‑GAGGAagtcgcacgacagaaataaattgaccgcgctaaggcccgtaaagaacgtcacgaggggcgc                                                                                                                                                                                                                                          <  1:704478‑M1/297‑62 (MQ=255)`  
                                                       `cGTGACACTGATGACCTGCACGATGCTATTCACATGGCTGCCGATAATGCAGTTCCGCACTACTACGCTGACATCTTTAGCGTAATGGCAAGTGAGGGCATTGACCTTGAGTTCGAAGACTCTGGTCTGATGCCTGACACCAAGGACGTAATCCGCATCCTGCAAGCGCGTATCTATGAGCAATTAACGATTGACCTCTGGGAAGACGCAGAAGACTTGCTCAATGAATACTTGGAGGAAGTCGAGGAagtcgcacgacagaaagaaattgaccgcgctaaggcccgtaaagaacgtcacg                                                                                                                                                                                                                                                  <  1:746175‑M1/301‑54 (MQ=255)`  
                                                           `acacTGATGACCTGCACGATGCTATTCACATGGCTGCCGATAATGCAGTTCCGCACTACTACGCTGACATCTTTAGCGTAATGGCAAGTGAGGGCATTGACCTTGAGTTCGAAGACTCTGGTCTGATTCCTGACACCAAGGACGTAATCCGCATCCTGCAAGCGCGTATCTATGAGCAATTAACGATTGACCTCTGGGAAGACGCAGAAGACTTGCTCAATGAATACTTG‑‑‑‑‑‑‑‑‑GAGGAagtcgcacgacagaaagaaattgaccgcgctaaggcccgtaaagactgtctcttatacacatctcc                                                                                                                                                                                                                                     >  1:57881‑M1/1‑235 (MQ=255)`  
                                                            `cacTGATGACCTGCACGATGCTATTCACATGGCTGCCGATAATGCAGTTCCGCACTACTACGCTGACATCTTTAGCGTAATGGAAAGTGAGGGCATTGACCTTGAGTTCGAAGACTCTGGTCTGATGCCTGACACCAAGGACGTAATCCGCATCCTGCAAGCGCGTATCTATGAGCAATTAACGATTGACCTCTGGGAAGACGCAGAAGACTTGCTCAATGAATACTTGGAGGAAGTCGAGGAagtcgcacgacagaaagaaattgaccgcgctaaggcccgtaaagactgtctcttatac                                                                                                                                                                                                                                             >  1:209143‑M1/1‑243 (MQ=255)`  
                                                              `cTGATGACCTGCACGATGCTATTCACATGGCTGCCGATAATGCAGTTCCGCACTACTACGCTGACATCTTTAGCGTAATGGCAAGTGAGGGCATTGACCTTGAGTTCGAAGACTCTGGTCTGATGCCTGACACCAAGGACGTAATCCGCATCCTGCAAGCGCGTATCTATGAGCAATTAACGATTGACCTCTGGGAAGACGCAGAAGACTTGCTCAATGAATACTTGGAGGAAGT‑‑‑‑‑gcacgacagaaagaaattgaccgcgctaaggcccgtaaagaacgtcacgaggggcgcttagaggc                                                                                                                                                                                                                                       >  1:383591‑M1/1‑236 (MQ=255)`  
                                                                  `tgaCCTGCACGATGCTATTCACATGGCTGCCGATAATGCAGTTCCGCACTACTACGCTGACATCTTTAGCGTAATGGCAAGTGAGGGCATTGAACTTGAGTTCGAAGACTCTGGTCTGATGCCTGACACCAAGGACGTAACCCGCATCCTGCAAGCGCGTATCTATGAGCAAGTAACGATTGACCTATGGGAAGACGCAGAAGACTTGCTCAATGAATAATTG‑‑‑‑‑‑‑‑‑GAGGAagtcgcacgacagaaagaaattgaccgcgctaaggcccgtaaagaacgtcacgaggggcgcttagaggcacgc                                                                                                                                                                                                                              <  1:214805‑M1/301‑74 (MQ=255)`  
                                                                     `ccTGCACGATGCTATTCACATGGCTGCCGATAATGCAGTTCCGCACTACTACGCTGACATCTTTAGCGTAATGGCAAGTGAGGGCATTGACCTTGAGTTCGAAGACTCTGGTCTGATGCCTGACACCAAGGACGTAATCCGCATCCTGCAAGCGCGTATCTATGAGCAATTAACGATTGACCTCTGGGAAGACGCAGAAGACTTGCTCAATGAATACTTGGAGGAAGTCGAGGAagtcgcacgacagaaagaaattgaccgcgctaaggcccgtaaagaacgtcacgaggggcgcttagag                                                                                                                                                                                                                                    <  1:329609‑M1/301‑68 (MQ=255)`  
                                                                       `tGCACGATGCTATTCACATGGCGGCCGATAATGCAGTTCCGCACTACTACGCTGACATCTTTAGCGTAATGGCAAGTGAGGGCATTGACCTGGAGTTCGAAGACTCTGGTCTGATGCCTGACACCAAGGACGTAATCCGCATCCTGCAAGCGCGTATCTATGAGCAATGAACGATTGACCTCTGGGAAGACGCAGAAGACTTGCTCAATGAATACTTGGAGGAAGTCGAGGAagtcgcacgacagaaagaaattgaccgcgttaaggcccgtaaagaacgtcacgaggggcgcttagaggc                                                                                                                                                                                                                                  <  1:525927‑M1/301‑70 (MQ=255)`  
                                                                           `agaTGCTAGTCACATGGCTGCCGATAATGCAGTTCCGAACTTCTACGCTGACATCTTTAGCGTAATGGCAAGGGAGGGCATTGACCTGGAGTTCGAAGACTCTGGTCTGATGCCTGACACCAAGGACGTAATCTGCATCCTGAAAGCGCGTGTCTATGAGCAAGTAACGATTGACCTCTGGGAAGACGCAGAAGACTTGCTCAATGAATTCTTGGAGGAAGTCGAGGAagtcgcacgacagaaagaaattgaccgagctaaggcccgtaaagaacgtcacgaggggcgcttagaggcacgc                                                                                                                                                                                                                              <  1:155443‑M1/300‑74 (MQ=255)`  
                                                                               `gCTATTCACATGGCTGCCGATAATGCAGTTCCGCACTACTACGCTGACATCTTTAGCGTAATGGCAAGTGAGGGCATTGACCTTGAGTTCGAAGACTCTGGTCTGATGCCTGACACCAAGGACGTAATCCGCATCCTGCAAGCGCGTATCTATGAGCAATTAACGATTGACCTCTGGGAAGACGCAGAAGACTTGCTCAATGAATACTTGGAGGAAGTCGAGGAagtcgcacgacagaaagaaattgaccgcgctaaggcccgtaaagaacgtcacgaggagcgcttagaggcacgcagat                                                                                                                                                                                                                          >  1:432141‑M1/1‑224 (MQ=255)`  
                                                                                `cTATTCACATGGCTGCCGATAATGCAGTTCCGCACTACTACGCTGACATCTTTAGCGTAATGGCAAGTGAGGGCATTGACCTTGAGTTCGAAGACTCTGGTCTGATGCCTGACACCAAGGACGTAATCCGCATCCTGCAAGCGCGTATCTATGAGCAATTAACGATTGACCTCTGGGAAGACGCAGAAGACTTGCTCAATGAATACTTGGAGGAAGTCGACGAagtcgcacgacagaaagaaattgaccgcgctaaggcccgtaaagaacgtcacgaggggcgcctgtctcttatacacat                                                                                                                                                                                                                         >  1:630240‑M1/1‑223 (MQ=255)`  
                                                                                 `tATTCTCATGGCTGCCGATAATGCAGTTCCGCACTACTACGCTGACATCTTTAGCGTAATGGCAAGTGAGGGCATTGACCTTGAGTTCGAAGACTCTGGTCTGATGCCTGACACCAAGGACGTAATCCGCATCCTGCAAGCGCGTATCTATGAGCAATTAACGATTGACCTCTGGGAAGACGCAGAAGACTTGCTCAATGAATACTTGGAGGAAGTCGAGGAagtcgcacgacagaaagaaattgaccgcgctaaggcccgtaaagaacgtcacgaggggagcttagaggcacgcagattc                                                                                                                                                                                                                        <  1:484357‑M1/301‑80 (MQ=255)`  
                                                                                 `gagatgtgtataagagacagatgcagttccgcactactacgCTGACATCTTTAGCGTAATGGCAAGTGAGGGCATTGACCTTGAGTTCGAAGACTCTGGTCTGATGCCTGACACCAAGGACGTAATCCGCATCCTGCAAGCGCGTATCTATGAGCAATTAACGATTGACATCTGGGAAGACGCAGAAGACTTGCTCAATGAATACTTGGAGGAAGTCGAGGAagtcgcacgacagaaagaaattgaccgcgctaaggcccgtaaagaacgtcacgaggggcgcttagaggcacgaagattc                                                                                                                                                                                                                        <  1:76123‑M1/281‑80 (MQ=255)`  
                                                                                  `aTTCACATGGCTGCCGATAATGCAGTTCCGCACTACTACGCTGACATCTTTAGCGTAATGGCAAGTGAGGGCATTGACCTTGAGTTCGAAGACTCTGGTCTGATGCCTGACACCAAGGACGTAATCCGCATCCTGCAAGCGCGTATCTATGAGCAATTAACGATTGACCTCTGGGAAGACGCAGAAGACTTGCTCAATGAATACTTGGAGGAAGTCGAGGAagtcgcacgacagaaagaaattgaccgcgctaaggcccgtaaagaacgtcactaggggcgcttagaggcacgcagattca                                                                                                                                                                                                                       >  1:240726‑M1/1‑221 (MQ=255)`  
                                                                                  `aTTCACATGGCTGCCGATAATGCAGTTCCGCACTACTACGCTGACATCTTTAGCGTAATGGCAAGTGAGGGCATTGACCTTGAGTTCGAAGACTCTGGTCTGATGCCTGACACCAAGGACGTAATCCGCATCCTGCAAGCGCGTATCTATGAGCAATTAACGATTGACCTCTGGGAAGACGCAGAAGACTTGCTCAATGAATACTTGGAGGAAGTCGAGGAagtcgcacgacagaaagaaattgaccgcgctaaggcccataaagaacgtaacgctgtctcttatacacatctccgatccc                                                                                                                                                                                                                       >  1:259032‑M1/1‑221 (MQ=255)`  
                                                                                  `aTTCACATGGCTGCCGATAATGCAGTTCCGCACTACTACGCTGACATCTTTAGCGTAATGGCAAGTGACGGCATTGACCTTGAGTTCGAAGACTCTGGTCTGATGCCTGACACCAAGGACGTAATCCGCATCCTGCAAGCGCGTATCTATGAGCAATTAACGATTGACCTCTGGGAAGACGCAGAAGACTTGCTCAATGAATACTTGGAGGCAGTCGAGGAagtcgcacgacagaacgaaattgaccacgctcaggcccgtcaagaacgtcccgaggctgtctcttatacacatctccgag                                                                                                                                                                                                                       >  1:225172‑M1/1‑221 (MQ=255)`  
                                                                                    `tCACATGGCTGCCGATAATGCAGTTCCGCACTACTACGCTGACATCTTTAGCGTAATGGCAAGTGAGGGCATTGACCTTGAGTTCGAAGACTCTGGTCTGATGCCTGACACCAAGGACGTAATCCGCATCCTGCAAGCGCGTATCTATGAGCAATTAACGATTGACCTCTGGGAAGACGCAGAAGACTTGCTCAATGAATACTTG‑‑‑‑‑‑‑‑‑GAGGAagtcgcacgacagaaagaaattgaccgcgctaaggcccgtaaagaacgtcacgaggggcgcttagaggcacgcagattcaaacgtcgcaac                                                                                                                                                                                                            >  1:465840‑M1/1‑210 (MQ=255)`  
                                                                                    `tCACATGGCTGCCGATAATGCAGTTCCGCACTACTACGCTGACATCTTTAGCGTAATGGCAAGTGAGGCCATTGACCTTGAGTTCGAAGACTCTGGTCTGATGCCTGACACCAAGGACGTAATCCGCATCCTGCAAGCGCGTATCTATGAGCAATTAACGATTGACCTCTGGGAAGACGCAGAAGACTTGCTCAATGAATACTTGGAGGAAGTCGAGTAagtcgcacgacagcaagcaattgaccgcgctaaggcccgtaaagaacgtcaagagctgtctcttatacacatctccgagccc                                                                                                                                                                                                                     >  1:563558‑M1/1‑219 (MQ=255)`  
                                                                                     `cacaTGGCTGCCGATAATGCAGTTCCGCACTACTACGCTGACATCTTTAGCGTAATGGCAAGTGAGGGCATTGACCTTGAGTTCGAAGACTCTGGTCTGATGCCTGACACCAAGGACGTAATCCGCATCCTGCAAGCGCGTATCTATGAGCAATTAACGATTGACCTATGGGAAGACGCAGAAGACTTGCTCAATGAATACTTGGAGGAAGTCGAGGAagtcgcacgacagaaagaaattgaccgcgctaaggcccgtaaagaacgtcacgaggggcgcttagaggcacgcagattcaaac                                                                                                                                                                                                                    <  1:373531‑M1/301‑84 (MQ=255)`  
                                                                                     `cacaTGGCTGCCGATAATGCAGTTCCGCACTACTACGCTGACATCTTTAGCGTAATGGCAAGTGAGGGCATTGACCTTGAGTTCGAAGACTCTGGTCTGATGCCTGACACCAAGGACGTAATCCGCATCCTGCAAGCGCGTATCTAAGAGCAATTAACGATTGACCTCTGGGAAGACGCAGAAGACTTGCTCAATGAATACTTGGAGGAAGTCGAGGAagtcgcccgacagaaagaaattgaccgcgctaaggcccgtaaagaacgtcacgaggggcgcttagaggcacgcagattcaaac                                                                                                                                                                                                                    <  1:740586‑M1/301‑84 (MQ=255)`  
                                                                                     `cacaTGGCTGCCGATAAGGCAGTTCCGCACTACTACGCTGACATCTTTAGCGTAATGGCAAGTGAGGGCATTGACCTTGAGTTCGAAGACTCTGGTCTGATGCCTGACACCAAGGACGTAATCCGCATCCTGCAAGCGCGTATCTATGAGCAATTAACGATTGACCTCTGTGAAGACGCAGAAGACTTGCTCAATGAATACTTGGAGGAAGTCGAGGAagtcgcacgacagaaagaaattgaccgcgctaaggcccgtaaagaacgtcactaggggcgcttagaggcacgcagattcaaac                                                                                                                                                                                                                    <  1:810463‑M1/301‑84 (MQ=255)`  
                                                                                     `cacaTGGCTGCCAATAATGCAGTTCCGCACAACTACGCTGACATCTTTATCGTAATGGCAAGTGAGGGCATTGACCTGGAGTTCGAAGACTCTGGTCTGATGCCTGACACCAAGGACGTAATCCGCATCCTGCAAGCGCGTATCTATGAGCAATTAACGATTGACCTCTGGGAAGACGCAGAAGACTTGCTCAATGAATACTTGGAGGAAGTCGAGGAagtcgcacgacagaaagaaattgaccgcgctaaggcccgtaaagaacgtcacgaggggcgcttagaggcacgcagattcaaac                                                                                                                                                                                                                    <  1:42640‑M1/301‑84 (MQ=255)`  
                                                                                             `tGCCGATAATGCAGTTCCGCACTACTACGCTGACATCTTTAGCGTAATGGCACGTGAGGGCATTGACCTTGAGTTCGAAGACTCTGGTCTGATGCCTGACACCAAGGACGTAATCCGCATCCTGCAAGCGCGTATCTATGAGCAATTAACGATTGACCTCTGGGAAGACGCAGAAGACTTGCTCAATGAATACTTGGAGGAAGTCGAGGAagtcgcacgacagaaagaaattgaccgcgctaaggcccgtaaagaacgtcacgaggggcgcttagaggcacgcagattcaaacgtcgcaac                                                                                                                                                                                                            <  1:103716‑M1/301‑92 (MQ=255)`  
                                                                                                     `gacaggttccgCACTACTACGCTGACATCTTTAGCGTAATGGCAAGTGAGGGCATTGACCTTGAGTTCGAAGACTCTGGTCTGATGCCTGACACCAAGGACGTAATCCGCATCCTGCAAGCGCGTATCTATGAGCAATTAACGATTGACCTCTGGGAAGACGCAGAAGACTTGCTCAATGAATACTTGGAGGAAGTCGAGGAagtcgcacgacagaaagaaattgaccgcgctaaggcccgtaaagaacgtcacgaggggcgcttagaggcacgcagattcaaacgtcgcaaccgcaaggc                                                                                                                                                                                                    <  1:517337‑M1/296‑100 (MQ=255)`  
                                                                                                     `aTGCAGTTCCGCACTACTACGCTGACATCTTTAGCGTAATGGCAAGTGAGGGCATTGACCTTGAGTTCGAAGACTCTGGTCTGATGCCTGACACCAAGGACGTAATCCGCATCCTGCAAGCGCGTATCTATGAGCAATTAACGATTGACCTCTGGGAAGACGCAGAAGACTTGCTCAATGAATACTTGGAGGAAGTCGAGGAagtcgcacgacagaaagaaattgaccgcgctaaggcccgtaaagaacgtcacgaggggcgcttagaggcacgcagattcaaacgacgcaaacgcaaggc                                                                                                                                                                                                    >  1:186762‑M1/1‑202 (MQ=255)`  
                                                                                                          `gTTCCTCACTACTACGCTGACATCTTTAGCGTAATGGCAAGTGAGGGCATTGACCTTGAGTTCGAAGACTCTGGTCTGATGCCTGACACCAAGGACGTAATCCGCATCCTGCAAGCGCGTATCTATGAGCAATTAACGATTGACCTCTGGGAAGACTCAGAAGACTTGTTCAATGAATACTTGGAGGAAGTCGAGGAagtcgcacgacagaaagaaattgaccgcgctaaggcccgtaaagaacgtcacgaggggcgcttagaggcacgcagattcaaacgtcgcaaccgcaaggcacgca                                                                                                                                                                                               >  1:622719‑M1/1‑197 (MQ=255)`  
                                                                                                           `atgggtataagagacaggacatctttagcgtaatgGCAAGTGAGGGCATTGACCTTGAGTTCGAAGACTCTGGTATGATGCCTGACACCAAGGACGTAATCCGCATCCTGCAAGCGCGTATCTATGAGCAATTAACGATTGACCTCTGGGAAGACGCAGAAGACTTGCTCAATGAATACTTGGAGGAAGTCGAGGAagtcgcacgacagaaagaaattgaccgcgctaaggcccgtaaagaacgtcacgaggggcgcttagaggcacgcagattcaaacgtcgcaaccgcaaggcacgtaa                                                                                                                                                                                              <  1:820058‑M1/284‑106 (MQ=255)`  
                                                                                                           `acaggaactactacgctgACATCTTTAGCGTAATGGCAAGTGAGGGCATTGACCTTGAGTTCGAATACTCTGGTCTGATGCCTGACACCAAGGACGTAATCCGCATCCTGCAAGCGCGTATCTATGAGCAATTAACGATTGACCTCTGGGAAGACGCAGAAGACTTGCTCAATGAATACTTGGAGGAAGTCGAGGAagtcgcacgacagaaagaaattgaccgcgctaaggcccgtaaagaacgtcacgaggggcgcttagaggcacgcagattcaaacgtcgcaaccgcaaggcacgtaa                                                                                                                                                                                              <  1:131305‑M1/295‑106 (MQ=255)`  
                                                                                                            `tCCGCACGACTACGCTGACATCTTTAGCGTAATGGCAAGTTAGGGCATCGACCTTGAGTTCGAAGACTCTGGTCTGATGCCTGACACCAAGGACGTAATCCGCATCCTGCAAGCGCGTATCTATGAGCAAGTAACGATTGACCTCTGGGAAGACGCAGAAGACTTGCTCAATGAATACTTGGAGGAAGTCGAGGAagtcgcacgacagaaagaaattgaccgcgctaaggcccgtaaagaacgtcacgaggggcgcttagaggcacgcagattcaaacgtcgcaaccgcaaggcacgtaaa                                                                                                                                                                                             <  1:604485‑M1/301‑107 (MQ=255)`  
                                                                                                                    `actacGCTGACATCTTTAGCGTAATGGCAAGTGAGGGCATTGACCTTGAGTTCGAAGACTCTGGTCTGATGCCTGACACCAAGGACGTAATCCGCATCCTGCAAGCGCGTATCTATGAGCAATTAACGATTGACCTCTGGGAAGACGCAGAAGACTTGCTCAATGAATACTTGGAGGAAGTCGAGGAagtcgcacgacagaaagaaattgaccgcgctaaggcccgtaaagaacgtcatgaggggcgcttagaggcacgcagattcaaacgtcgctgtctcttatacacatctccgagccc                                                                                                                                                                                     >  1:127426‑M1/1‑187 (MQ=255)`  
                                                                                                                    `accacgctGACATCTTTAGCGTAATGGCAAGTGAGGGCATTGACCTTGAGTTCGAAGACTCTGGTCTGATGCCTGACACCAAGGACGTAATCCGCATCCTGCAAGCGCGTATCTATGAGCAATTAACGATTGACCTCTGGGAAGACGCAGAAGACTTGCTCAATGAATACTTGGAGGAAGTCGAGGAagtcgcacgacagaaagaaattgaccgcgctaaggcccgtaaagaacgtcacgagtggcgcttagaggcacgcagattcaaacgtcgcaaccgcaaggcccgtaaagcacacaa                                                                                                                                                                                     >  1:698509‑M1/4‑187 (MQ=255)`  
                                                                                                                       `acGCTGACATCTTTAGCGTAATGGCAAGTGAGGGCATTGACCTTGAGTTCGAAGACTCTGGTCTGATGCCTGACACCAAGGACGTAATCCGCATCCTGCAAGCGCGTATCTATGAGCAATTAACGATTGACCTCTGGGAAGACGCAGAAGACTTGCTCAATGAATACTTGGAGGAAGTCGAGGAagtcgcacgacagaaagaaattgaccgcgctaaggcccgtaaagaacgtcacgaggggcgcttagaggcacgcagattcaaacgtcgcaaccgcaaggcacgtaaagcacacaaagc                                                                                                                                                                                  <  1:307620‑M1/301‑118 (MQ=255)`  
                                                                                                                       `acGCTGACATCTTTAGCGTAATGGCAAGTGAGGGCATTGACCTTGAGTTCGAAGACTCTGGTCTGATGCCTGACACCAAGGACGTAATCCGCATCCTGCAAGCGCGTATCTATGAGCAATTAACGATTGACCTCTGGGAAGACGCAGAAGACTTGCTCAATGAATACTTGGAAGAAGTCGAGGAagtcgcacgacagaaagaaattgaccgcgctaaggcccgtaaagaacgtcacgaggggcgcttagaggcacgcagattcaaacgtcgcaaccgcaaggcacgtaaagcacacaaagc                                                                                                                                                                                  >  1:390333‑M1/1‑184 (MQ=255)`  
                                                                                                                          `cTGACATCTTTAGCGTAATGGCAAGTGAGGGCATTGACCTTGAGTTCGAAGACTCTGGTCTGATGCCTGACACCAAGGACGTAATCCGCATCCTGCAAGCGCGTATCTATGAGCAATTAACGATTGACCTCTGGGAATACGCAGAAGACTTGCTCAATGAATACTTGGAGGAAGT‑‑‑‑‑gcacgacagaaagaaattgaccgcgctaaggcccgtaaataacgtcacgaggggcgcttagaggcacgcagattcaaacgtcgcaaccgcaaggcacgtaaagcacacaaagctaagcgcgaaag                                                                                                                                                                           >  1:280144‑M1/1‑176 (MQ=255)`  
                                                                                                                              `cATCTTTAGCGTAATGGCAAGTGAGGGCATTGACCTTGAGTTCGAAGACTCTGGTCTGATGCCTGACACCAAGGACGTAATCCGCATCCTGCAAGCGCGTATCTATGAGCAATTAACGATTGACCTCTGGGAATACGCAGAAGACTTGCTCAATGAATACTTGGAGGAAGTCGAGGAagtcgcacgacagaaagaaattgaccgcgctaaggcccgtaaagaacgtcacgaggggcgcttataggcacgcagattcaaacgtcgcaaccgcaaggcacgtaaagcacacaaagctaagcgc                                                                                                                                                                           >  1:769808‑M1/1‑177 (MQ=255)`  
                                                                                                                                 `cTTTAGCGTAATGGCAAGTGAGGGCATTGACCTTGAGTTCGAAGACTCTGGTCTGATGCCTGACACCAAGGACGTAATCCGCATCCTGCAAGCGCGTATCTATGAGCAATTAACGATTGACCTCTGGGAAGACGCAGAAGACTTGCTCAATGAATACTTGGAGGAAGTCGAGGAagtcgcacgacagcaagaaattgaccgcgctaaggcccgtaaagaacgtcacgaggggcgcttagaggcacgcagattcaaacgtcgcaaccgcaaggcacgtaaagcacacaaagctaagcgcgaa                                                                                                                                                                        >  1:136778‑M1/1‑174 (MQ=255)`  
                                                                                                                                   `ttggcgtAATGGCAAGTGAGGGCATTGACCTTGAGTTAGAAGACTCTGGTCTGATGCCTGAAACAAAGGACGTAATCCGCAGCCTGCAAGCGCGTATCTATGAGCAATTAACGATTGACAGCTGGGAAGACGCAGAAGACTTGCTCAATGAATACTTGGAGGAAGTCGAGGAaggagcacgacagaaagaaattgaccgcgctaaggcccgtaaagaacgtcacgaggggcgcttagaggcacgcagattcaaacgtcgcaaccgcaaggcacgtaaagcacacaaagctaagcgcaaaag                                                                                                                                                                      <  1:3425‑M1/298‑130 (MQ=255)`  
                                                                                                                                         `tAATGGCAATTGATGGCATTGACCTTGAGTTCGAATACTCTGGTCTGATGCCTGACACCAAGGACGTAATCCGCATCCTGCAAGCTCGTATCTATGAGCAATTAACGATTGACCTCTGTGAAGACGCAGAAGACTTGCTCAATGAATACTTGGAGGAAGTCGAGGAagtcgcacgacagaaagaaattgaccgcgctaaggcccgtaaagaacgtcacgaggggcgcttagaggcacgcagattcaaacgtcgcaaccgcaaggcacgtaaagcacacaaagctaagcgcgaaagaatgct                                                                                                                                                                <  1:716711‑M1/301‑136 (MQ=255)`  
                                                                                                                                         `tAATGGCAAGTGAGGGCATTGACCTTGAGTTCGAAGACTCTGGTCTGATGCCTGACACCAAGGACGTAATCCGCATCCTGCAAGCGCGTATCTATGAGCAATTAACAATTGACCTCTGGGAAGACGCAGAAGCCTTGCTCAATGAATACTTGGAGGCAGTCGAGGAagtcgcacgacagaaagaaattgaccgcgctaaggcccgtaaagaacgtcacgaggggcgcttagaggcacgcacatacaaacgtcgcaaccgcaaggcacgtaaagcacacaaagctaagcgcgaaagcatgct                                                                                                                                                                >  1:668820‑M1/1‑166 (MQ=255)`  
                                                                                                                                                  `ataagagacaggaccttgagttcGAAGACTCTGGTCTGATGCCTGACACCAAGGACGTAATCCGCATCATGCAAGCGCGTATCTATGAGCAATTAACGATTGACCTCTGGGAAGACGCAGAAGACTTGCTCAATGAATACTTG‑‑‑‑‑‑‑‑‑GAGGAagtcgcacgacagaaagaaattgaccgcgctaaggcccgtaaagaacgtcacaaggggcgcttagaggcacgcagattcaaacgtcgcaaccgcaaggcacgtaaagcacacaaagctaagcgcgaaagaatgcttgctgcgtggcgatgggc                                                                                                                                              <  1:288765‑M1/290‑154 (MQ=255)`  
                                                                                                                                                             `gACCTTGAGTTCGAAGACTCTGGTCTGATGCCTGACACCAAGGACGTAATCCGCATCCTGCAAGCGCGTATCTATGAGCAATTAACGATTGACCTCTGGGAAGACGCAGAAGACTTGCTCAATGAATACTTGGAGGAAGTCGAGGAagtcgcacgacagaaagaaattgaccgcgctaaggcccgtaaagaacgtcacgaggggcgcttagaggcacgcagattcaaacgtcgcaaccgcaaggcacgtaaagcacacaaagctaagcgcgaaagaatgcttgctgcgtggcgatgggctg                                                                                                                                            >  1:362200‑M1/1‑146 (MQ=255)`  
                                                                                                                                                               `ccTTGAGTTCGAAGACTCTGGTCTGATGCCTGACACCAAGGACGTAATCCGCATCCTGCAAGCGCGTATCTATGAGCAATTAACGATTGACCTCTGGGAAGACGCAGAAGACTTGCTCAATGAATACTTGGAGGAAGTCGAGGAagtcgcacgacagaaagaaattgaccgcgctaaggcccgtaaagaacgtcacgaggggcgcttataggcacgcagattcaaacgtcgcaaccgcaaggcacgtaaagcacacaaagctaagcgcgaaagaatgcttgctgcgtggcgatgggctgaa                                                                                                                                          >  1:264284‑M1/1‑144 (MQ=255)`  
                                                                                                                                                                              `ctctGGTCTGATGCCTGACACCAAGGACGTAATCCGCATCCTGCAAGCGCGTATCTATGAGCAATTAACGATTGACCTCTGGGAAGACGCAGAAGACTTGCTCAATGAATACTTGGAGGAAGTCGAGGAagtcgcacgacagaaagaaattgaccgcgctaaggcccgtaaagaacgtcacgaggggcgcttagaggcacgcagattcaaacgtcgcaaccgcaaggaacgtaaagcacacaaagctaagcgcgaaagaatgcttgctgcgtggcgatgggctgaacgtcaagaacggcgt                                                                                                                           >  1:796080‑M1/1‑129 (MQ=255)`  
                                                                                                                                                                               `aagtgtctgATGCCTGACACAAAGGACGTAATCCGCATCCTGCAAGCGCGTATCTAAGAGCAATTAACGATTGACCTCTGGGAAGACGCAGAAGACTTGCTCAATGAATACTTG‑‑‑‑‑‑‑‑‑GAGGAagtcgcacgacagaaagaaattgaccgcgctaaggcccgtaaagaacgtcacgaggggcgcttagaggcacgcagattcaaacgtcgcaaccgcaaggcacgtaaagcacacaaagctaagcgcgaaagaatgcttgctgcgtggcgatgggctgaacgtcaagaacggcgtaaccatgagg                                                                                                                 <  1:539298‑M1/297‑183 (MQ=255)`  
                                                                                                                                                                                 `cggactgagGTCTGACACCAAGGACGTAATCCGCATCCTGCAAGCGCGTATCTATGAGCAATTAACGTTTGACCTCTGGGAAGACGCAGAAGACTTGCTCAATGAATACTTGGAGGAAGTCGAGGAagtcgcacgacagaaagaaattgaccgcgctaaggcccgtaaagaacgtcacgaggggagcttagaggcacgcagattcaaacgtcgcaaccgcaaggcacgtaaagcacacaaagctaagcgcgaaagaatgcttgctgcgtggcgatgggctgaacgtcaagaacggcggaac                                                                                                                        <  1:601237‑M1/297‑176 (MQ=255)`  
                                                                                                                                                                                  `ggTCTGATGCCTGACACCAATGACGTAATCCGCATCCTGCAAGCGCGTATCTATGAGCAATTAACGATTGACCTCTGGGAAGACGCAGAAGACTTGCTCAATGAATACTTGGATGAAGTCGAGGAagtcgcacgacagaaagaaattgaccgcgctaaggcccgtaaagaacgtcacgaggggcgcttagaggcacgcagattcaaacgtcgcaaccgcaaggcacgtaaagcatacaaagctaagcgcgaaagaatgcttgctgcgtggcgatgggctgaacgtcaagaacggcgtaacc                                                                                                                       >  1:141847‑M1/1‑125 (MQ=255)`  
                                                                                                                                                                                  `ggTCTGATGCCTGACACCAAGGACGTAATCCGCATCCTGCAAGCGCGTATCTATGAGCAATGAACGATTGACCTCGGGGAAGACGCAGAAGACTTGCTCAATGAATACTTGGAGGAAGTCGAGGAagtcgcacgacagaaagaaattgaccgctctaaggcccgtaaagaacgtcacgaggggcgcttagaggcacgcagattcaaatgtagcaaccgcaaggcacgtaaagcacacaaagctaagcgcgaaagaatgcttgctgcgtggcgatgggctgaacgtcaagaacggcgtaaac                                                                                                                       >  1:709715‑M1/1‑125 (MQ=255)`  
                                                                                                                                                                                       `gATGCCTGACACCAAGGACGTAATCCGCATCCTGCAAGCGCGTATCTATGAGCAATTAACGATTGACCTCTGGGAAGACGCAGAAGACTTGCTCAATGAATACTTGGAGGAAGTCGAGGAagtcgcccgacagaaagaaattgaccgcgctaaggcccgtaaagcacgtcacgaggggcgcttagaggcacgcagattcaaacgtcgcaaccgaaaggcacgtaaagcttacaaagctaagcgcgaaagcatgcttcccgcgtggcgatgggctgacagtccagaacggcttaaccatgag                                                                                                                  >  1:498516‑M1/1‑120 (MQ=255)`  
                                                                                                                                                                                        `aTGCCTGACACCAAGGACGTAATCTGCATCCTGCAAGCGCGTATCTATGAGCAATTAACGATTGACCTCTGGGAAGACGCAGAAGACTTGCTCAATGAATACTTGGAGGAAGTCGAGGAagtcgcacgacagaaagaaattgaccgcgctaaggcccgtaaagaacgtcacgaggggcgcttagaggcacgcagattcaaacgtcgcaaccgcaaggcacgtaaagcacacaaagctaagcgcgaaagaatgcttgctgcgtggcgatgggctgaacgtcaagaacggcgtaaccatgagg                                                                                                                 <  1:203877‑M1/301‑183 (MQ=255)`  
                                                                                                                                                                                          `gCCTGACACCAAGGACGTAATCCGCATCCTGCAAGCGCGTATCTATGAGCAATTAACGATTGACCTCTGGGAAGACGCAGAAGACTTGCTCAATGAATACTTGGAGGAAGTCGAGGAagtcgcacgacagaaagaaattgaccgcgctaaggcccgtaaagaacgtcacgaggggcgcttagaggcacgcagattcaaacatcgcaaccgcaaggcacgtaaagcacacaaagctaagcgcgaaagaatgcttgctgcgtggcgatgggctgaacgtcaagaacggcgtaaccatgaggta                                                                                                               >  1:78886‑M1/1‑117 (MQ=255)`  
                                                                                                                                                                                           `ccTGACACCAAGGACGTAATCCGCATCCTGCAAGCGCGTATCTATGAGCAATTAACGATTGACCTCTGGGAAGACGCAGAAGACTTGCTCAATGCATACTTGGAGGAAGTCGAGGAagtcgcacgacagaaagaaattgaccgcgctaaggcccgtaaagaacgtcacgaggggcgcttagaggcacgcagattcaaacgtcgcaaccgcaaggcacgtaaagcacacaaagctaagcgcgaaagaatgcttgctgcgtggcgattggctgaacgtcaagaacggcgtaaccatgaggtag                                                                                                              >  1:630552‑M1/1‑116 (MQ=255)`  
                                                                                                                                                                                              `gACACCAAGGACGTAAGCCGCATCCGGCAAGCGCGTATCTATGAGCAATTAACGATTGACCTCTTGGAAGACGCAGAAGACTTGCTCAATGAATACTTGGAGGAAGTCGAGGAagtcgcacgacagaaagaaattgaccgcgctaaggcccgtaaagaacgtcacgaggggcgcttagaggcacgcagattcaaacgtcgcaaccgcaaggcacgtaaagcacacaaagctaagcgcgaaataatgcttgctgcgtggcgatgggctgaacgtcaagaacggcgtaaccatgaggtagctg                                                                                                           <  1:164218‑M1/301‑189 (MQ=255)`  
                                                                                                                                                                                              `aacaccAAGGACGTAATCCGCAACCTGCAAGGGCGGATCTAAGAGCAATTAACGATTGAACTCTTGGAAGACGAAGAAGAATTGCTAAATGAATACTTG‑‑‑‑‑‑‑‑‑GAGGAagtcgcacgaaagaaagaaattgaccgggctaaggcccgtaaagaacgtcacgaggggcgcttagaggcacgcagattcaaacgtcgcaaccgcaaggcacgtaaagcacacaaagctaagcgcgaaagaatgcttgctgcgtggcgatgggctgaacgtcaagaacggcgtaaccatgaggtagctgtagatgtac                                                                                                  <  1:610668‑M1/300‑198 (MQ=255)`  
                                                                                                                                                                                                `gagacatgccctaatccgcatccTGCAAGCGCGTTTCTATGAGCAATTAACGATTGACCTCTGGGAAGACGCAGAAGACTTGCTCAATGAATACTTG‑‑‑‑‑‑‑‑‑GAGGAagtcgcacgacagaaagaaattgaccgcgctaaggcccgtaaagaacgtcacgatgggcgcttagaggcacgcagattcaaacgtcgcaaccgcaaggcacgtaaagcacacaaagctaagcgcgaaataatgcttgctgcgtggcgatgggctgaacgtcaagaacggcgtaaccatgaggtagctgtagatgtacta                                                                                                <  1:651576‑M1/290‑200 (MQ=255)`  
                                                                                                                                                                                                `cacCAAGGACGTAATCCGCATCCTGCAAGCGCGTATCTATGAGCAATTAACGATTGACCTCTGGGAAGACGCAGAAGACTTGCTCAATGAATACTTGGAGGAAGTCGAGGAagtcgcacgacagaaagcaattgaccgcgctaaggcccgtaaagaacgtcattaggggcgcttcgaggcacgcagattcaaacgtcgcaaccgcaaggcacgtaaagcacacacagctaagcgcgaaagaatgcttgctgcgtggcgatgggctgaacgtcaagaacggcgtaaccatgaggtagctgtt                                                                                                         >  1:801164‑M1/1‑111 (MQ=255)`  
                                                                                                                                                                                                   `gctcggagatgtgtataagagacaggcgcgtatctatgagcaattaacgattgaCCCCTGGGAAGACGCAGAAGACTTGCTCAATGAATACTTGGAGGA‑‑‑‑‑‑‑Gtcgcacgacagaaagaaattgaccgcgctaaggcccgtaaagaacgtaacgaggggcgcttagaggcacgcagattcaaacgtcgcaaccgcaaggcacgtaaagcacacaaagctaagcgcgaaagaatgcttgctgcgtggcgatgggctgaacgtcaagaacggcgtaaccatgaggtagctgtagatgtactagga                                                                                                <  1:323615‑M1/276‑201 (MQ=255)`  
                                                                                                                                                                                                   `aggtgtataagagacagtcctgcaagcgcgtatctATGAGCAATTAACGATTGACCTCTGGGAAGACGCAGAAGACTTGCTAAATGAATACTTGGAGGAAGTCGAGGAaggcgcacgacagaaagaaattgaccgcgctaaggcccgtaaagaacgtcacgaggggcgcttagaggcacgcagattcaaacgtcgcaaccgcaaggcacgtaaagcacacaaagctaagcgcgaaagaatgcttgctgcgtggcgatgggctgaacgtcaagaacggcgtaaccatgaggtagctgtagat                                                                                                      <  1:676738‑M1/284‑194 (MQ=255)`  
                                                                                                                                                                                                    `aaGGACGGAATCTGCATCCTGCAAGCGCGTATCTATGAGCAATTAACGATTGACCTCTGGGAAGACTCAGAAGACTTGCTCAATGAATACTTGGAGGAAGTCGAGGAagtcgaacgacagaaagaaattgaccgcgctaaggcccgtaaagaacgtcacgaggggcgcttagaggcacgcagattcaaacgtcgcaaccgcaaggcacgtaaagcacacaaagctaagcgcgaaagaatgcttgctgcgtggcgatgggctgaacgtcaagaacggcgtaaccatgaggtagctgtagatg                                                                                                     <  1:181721‑M1/301‑195 (MQ=255)`  
                                                                                                                                                                                                      `ggACGTAATCCGCATCCTGCAAGTGCGTATCTATGAGCAATTAACGATTGACCTCTGGGAAGACGCAGAAGACTTGCTCAATGAATACTTGGAGGAAGTCGAGGAagtcgcacgacagaaagaaattgaccgcgctaaggcccgtaaagaacgtcacgaggggcgcttagaggcacgcagattcaaacgtcgcaaccgcaaggcacgtaaagcacacaaagctaagcgcgaaagaatgcttgctgcgtggcgatgggctgaacgtcaagaacggcgtaaccatgaggtagctgtagactgt                                                                                                   >  1:638659‑M1/1‑105 (MQ=255)`  
                                                                                                                                                                                                       `gACGTAATCCGCATCCTGCAAGCGCGTATCTATGAGCAATTAACGATTGACATCTGGGAAGACGCAGAAGACTTGCTCAATGAATACTTGGAGGAAGTCGAGGAagtcgcacgacagaaagaaattgaccgcgctaaggcccgtaaagaacgtcacgaggggcgcttagaggcacgcagattcaaacgtcgcaaccgcaaggcacgtaaagcacacaaagctaagcgcgaaagaatgcttgctgcgtggcgatgggctgaacgtcaagaacggcgtaaccatgaggtagctgtagatgtac                                                                                                  <  1:574928‑M1/301‑198 (MQ=255)`  
                                                                                                                                                                                                            `aaTCCGCATCCTGCAAGCGCGTATCTATGAGCAATTAACGATTGACCTCTGTGAAGACGCAGAAGACTTGCTCAATGAATACTTGGAGGAAGTCGAGGAagtcgcacgacagaaagaaattgaccgcgctaaggcccgtaaagaacgtcactatgggcgcttagaggcacgcagattcaaacgtcgcaaccgcaaggcacgtaaagcacacaaagctaagcgcgaaagaatgcttgctgcgtggcgatgggctgaacgtcaagaacggcgtaaccatgaggtagctgtagatgtactagga                                                                                             <  1:337302‑M1/301‑203 (MQ=255)`  
                                                                                                                                                                                                            `aaTCCGCATCCTGCAAGCGCGTATCTATGAGCAATTAACGATTGACCTCTGGGAAGACGCAGAAGACTTGCTCAATGAATACTTGGAGGAAGTCGAGGAagtcgcacgacagaaagaaattgacctcgctaaggcccgtaaagaacgtcacgaggggcgcttagaggcacgcagattcaaacgtcgcaaccgcaaggcacgtaaagcacacaaagctaagcgcgaaagaatgcttgctgcgtggcgatgggctgaacgtcaagaacggcgtaaccatgaggtagctgtagatgtactagga                                                                                             <  1:591175‑M1/301‑203 (MQ=255)`  
                                                                                                                                                                                                            `aaTCCGCATCCTGCAAGCGCGTATCTATGAGCAATTAACGATTGACCTCTGGGAAGACGCAGAAGACTTGCTCAATGAATACTTGGAGGAAGTCGAGGAagtcgcacgacagaaagaaattgaccgcgctaaggcccgtaaagaacgtcacgaggggcgcttagaggcacgcagattcaaacgtcgcaaccgcaaggcacgtaaagcacacaaagctaagcgcgaaagaatgcttgctgcgtggcgatgggctgaacgtcaagaacggcgtaaccatgaggtctgtctcttatacacatct                                                                                             >  1:43584‑M1/1‑99 (MQ=255)`  
                                                                                                                                                                                                            `aaTCCGCATCCTGCAAGCGCGTATCTATGAGCAATTAACGATTGACCTCTGGGAAGACGCAGAAGACTTGCTCAATGAATACTTGGAGGAAGTCGAGGAagtcacacgacagaaagaaattgaccgcgctaaggcccgtaaagaacgtcacgaggggcgcttagaggcacgcagattcaaacgtcgcaaccgcaaggcacgtaaagcacacaaagctaagcgcgaaagaatgcttgctgcgtggcgatgggctgaacgtcaagaacggagtaaccatgaggtagctgtagatgtactagga                                                                                             <  1:327353‑M1/301‑203 (MQ=255)`  
                                                                                                                                                                                                              `tCCGCATCCTGCAAGCGCGTATCTATGAGCAATTAACGATTGACCTCTTGGACGACGCAGAAGACTTGCTCAATGAATACTTGGAGGAAGTCGAGGAagtcgcacgacagaaagaaattgaccgcgctaaggcccgtaaagaacgtcacgaggggcgcttagcggcacgcagattcaaacgtcgcaaccgcaaggcacgtacagcacacaacgctaagcgcgaaagaatgcttgctgcgtggcgatgggctgaacgtcaagaacggagtaatcatgaggtagctgtagaggtactaggaag                                                                                           >  1:596330‑M1/1‑97 (MQ=255)`  
                                                                                                                                                                                                                 `atgtgtataagagacagatctatgagcaattaacgATTGACCTCTGGGAAGACGCAGAAGACTTGCTCAATGAATAATTG‑‑‑‑‑‑‑‑‑GAGGAagtcgcacgacagaaagaaattgaccgcgctaaggcccgtaaagaacgtcacgaggggcgcttagaggcacgcagattcaaacgtcgcaaccgcaaggcacgtaaagcacacaaagctaagcgcgaaagaatgcttgctgcgtggcgatgggctgaacgtcaagaacggcgtaaccatgaggtagctgtagatgtactaggaagaaccaataacgc                                                                               <  1:823232‑M1/284‑217 (MQ=255)`  
                                                                                                                                                                                                                      `cTGCAAGCGCGTATCTATGAGCAATTAACGATTGACCTCTGGGAAGACGCAGAAGACTTGCTCAATGAATACTTG‑‑‑‑‑‑‑‑‑GAGGAagtcgcactacagaaagaaattgaccgcgctaaggcccgtaaagaacgtcacgatgggcgcttagaggcacgcagattcaaacgtcgcaaccgcaaggcacgtaaagcacacaaagctaagcgcgaaagaatgcttgctgcgtggcgatgggctgaacgtcaagaacggcgtaaccatgaggtagctgtcgatctgtctcttatacacatctccgcgcccc                                                                          >  1:271894‑M1/1‑80 (MQ=255)`  
                                                                                                                                                                                                                       `tataagagacagtctatgagcaattAACGATTGACCTCTGGGAAGACGCAGAAGACTTGCTCAATGAATACTTGGAGGAAGTCGAGGAagtcgcactacagaaagaaattgaccgcgctaaggcccgtaaagaacgtcactaggggcgcttagaggcacgcagattcaaacgtcgcaactgcaaggcacgtaaagcacacaaagctaagcgcgaaagaatgcttgctgcgtggcgatgggctgaacgtcaagaacggcgtaaccatgaggtagctgtagatgtactaggaagaaccaataa                                                                                  <  1:77387‑M1/289‑214 (MQ=255)`  
                                                                                                                                                                                                                        `ggaagcGCGTATCTATGAGCAATTAACGATTGACCTCTGGGAAGACGCAGAAGACTTGCTCAATGAATACTT‑‑‑‑‑‑‑‑T‑GAGGAagtcgcacgacagaaagaaattgaccgcgctaaggcccgtaaagaacgtcactaggggcgcttagaggcacgcagattcaaacgtcgcaaccgcaaggcacgtaaagcacacaaagctaagcgcgaaagaatgcttgctgcgtggcgatgggctgaacgtcaagaacggcgtaaccatgaggtagctgtagatgtactaggaagaaccaataacgctatgctc                                                                        <  1:246698‑M1/299‑224 (MQ=255)`  
                                                                                                                                                                                                                          `aaGCGCGTATCTATGAGCAATTAACGATTGACCTCTGGGAAGACGCAGAAGACTTGCTCAATGAATACTTGGAGTAAGTCGAGGAagtcgcacgacagaaagaaattgaccgcgctaaggcccgtaaagaacgtcacgaggggcgcttagaggcacgcagattcaaacgtcgcaaccgcaaggcacgtaaagcacacaaagctaagcgcgaaagaatgcttgctgcgttgcgatgggctgaacgtcaagaacggcgtaaccatgaggtagctgtagatgtactaggaagaacaaataacgc                                                                               <  1:659719‑M1/301‑217 (MQ=255)`  
                                                                                                                                                                                                                            `gcgcGTATCTATGAGCAATTAACGATTGACCTCTGGGAAGACGCAGAAGACTTGCTCAATGAATACTTGGAGGAAGTCGATGAagtcgcacgacagaaagaaattgaccgcgctaaggcccgtaaagaacgtcacgaggggcgcttagaggcacgcagattcaaacgtcgcaaccgcacggcacgtaaagcacacaaagctcagcgcgaaagaatgcttgctgcgtggcgatgggcttaacgtcccgaacggcgtaaccatgaggtagctgtagaggtactaggactgtctcttatacaca                                                                             >  1:725604‑M1/1‑83 (MQ=255)`  
                                                                                                                                                                                                                              `gcgTATCTATGAGCAATTAACGATTGACCTCTGGGAAGACGCAGAAGACTTGCTCAATGAATACTTGGAGGAAGTCGAGGAagtcgcacgacagaaagaaattgaccgcgctaaggcccgtaaagaacgtcacgatgggcgcttagaggcacgcagattcaaacgtcgcaaccgcaaggcacgtaaagcacacaaagctaagcgcgaaagaatgcttgctgcgtggcgatgggctgaacgtcaagaacggcgtaaccatgaggtagctgtagatgtactaggactgtctcttatacacatc                                                                           >  1:371309‑M1/1‑81 (MQ=255)`  
                                                                                                                                                                                                                               `cgTATCTATGAGCAATTAACGATTGACCTCTGGGAAGACGCAGAAGACTTGCTCAATGAATACTTGGAGGAAGTCGAGGAagtcgcacgacagaaagaaattgaccgcgctaaggcccataaagaacgtcacgaggggcgcttagaggcacgcagattcaaacgtcgcaaccgcaaggcacgtaaagcacacaaagctaagcgcgaaagaatgcttgatgcgtggcgatgggctgaacgtcaagaacggcgtaaccatgaggtagctgtagatgtactaggaagaaccaataacgctatgc                                                                          <  1:635146‑M1/301‑222 (MQ=255)`  
                                                                                                                                                                                                                                 `tATCTATGAGGAATTAACGATTGACCTCTGGGAAGACGCAGAAGACTTGCTCAATGAATACTTGGAGGAAGTCTAGGAagtcgcacgacagaaagaaattgaccgcgctaaggcccgtaaagaacgtcacgagtggcgcttagaggcacgcagattcaaacgtcgcaaccgcaaggcacgtaaagcacacaaagctaagcgcgaaagaatgcttgctgcgtggcgatgggctgaacgtcaagaacggcgtaaccatgaggtagctgtagatgtactaggaagaaccaataacactatgctc                                                                        <  1:172719‑M1/301‑224 (MQ=255)`  
                                                                                                                                                                                                                                 `tATCTATGAGCAATTAACGATTGACTTCTGGGAAGACGCAGAAGACTTGCTCAATGAATACTTG‑‑‑‑‑‑‑‑‑GAGGAagtcgcacgacagaaagaaattgaccgcgctaaggcccgtaaagaacgtcacgaggggcgcttagaggcacgcagattcaaacgtcgcaaccgcaaggcacgtaaagcacacaaagctaagcgcgaaagaatgcttgctgcgtggcgatgggctgaacgtcaagaacggcgtaaccatgaggtagctgtagatgtactaggaagaaccaataacgctatgctctgggtcaac                                                               <  1:303943‑M1/301‑233 (MQ=255)`  
                                                                                                                                                                                                                                 `tATCTATGAGCAATTAACGATTGACCTGTGGGAAGACGCAGAAGACTTGCTCAATGAATACTTGGAGGAAGTCGAGGAagtcgcacgacagaaagaaattgaccgcgctaaggcccgtaaagaacgtcacgaggggcgcttagaggcacgcagattcaaacgtcgcaaccgcaaggcacgtaaagcacacaaagctaagcgcgaaagaatgcttgctgcgtggcgatgggctgaacgtcaagaacggcgtaaccatgaggtagctgtagatgtactaggaagaaccaataacgctatgctc                                                                        <  1:733024‑M1/301‑224 (MQ=255)`  
                                                                                                                                                                                                                                 `tATCTATGAGCAATTAACGATTGACCTCTGGGAAGACGCAGAAGACTTGCTCAATGAATACTTGGAGGAAGTCGAGGAagtcgcacgacagaaagaaattgaccgggctaaggcccgtaaagaacgtcacgaggggcgcttagaggcacgcagattcaaacgtcgcaaccgcaaggcacgtaaagcacacaaagctaagcgcgaaagaatgcttgctgcgtggcgatgggctgaacgtcaagaacggcgtaaccatgaagtagctgtagatgtactaggaagaaccaataacgatatgctc                                                                        <  1:119167‑M1/301‑224 (MQ=255)`  
                                                                                                                                                                                                                                   `tCTATGAGCAATTAACGATTGACCTCTGGGAAGACGCAGAAGACTTGCTCAATGAATACTTGGAGGAAGTCGAGGAagtcgcacgacagaaagaaattgaccgcgctaaggcccgtaaagaacgtcacgaggggcgcttagaggcacgcagattcaaacgtcgcaaccgcaaggcacgtaaagcacacaaagctaagcgcgaaagaatgcttgctgcgtggcgatgggctgaacgtcaagaacggcgtaaccattaggtagctgtagatgtactaggaagaacccataacgctatgctctg                                                                      >  1:82643‑M1/1‑76 (MQ=255)`  
                                                                                                                                                                                                                                           `agattAATGATTGACCTCTGGGAAGACGCAGAAGACTTGCTCAATGAATACTTGGAGGAAGTCGAGGAagtcgcacgacagaaagaaattgaccgcgctaaggcccgtaaagaacgtcacgaggggcgcttagaggcacacagattcaaacgtcgcaaccgcaaggcacgtaaagcacacaaagctaagcgcgaaagaatgcttgctgcgtggcgatgggctgaacgtcaagaacggcgtaaccatgaggtagctgtagatgtactaggaagaaccaataacgctatgctctgagtcaaca                                                              <  1:474517‑M1/299‑234 (MQ=255)`  
                                                                                                                                                                                                                                                `accgaTTGGCCTCTGAGAAAACGCAGAAAACTTGTTCAATGAATACTTGGAGGAAGTATAGGAagttgcacgacagaaagaaatctaccgcgctaaggcccgtaaataacggcacgagtggcgctgagaggcacgcatattcaaacgtcgcaaccgcaaggcacgtaaagaacacaaagctaagcgcgaaagaatgcttgctgagtggcgatgggatgaacttcaagaacggcgtaaccatgaggtagctgtagatgtactaggaataaccaataacgctatgctctgggtcaacatgttc                                                         <  1:195180‑M1/299‑239 (MQ=255)`  
                                                                                                                                                                                                                                                `aaCGATTGACCTCTGGGAAGACGCAGAAGACTTGCTCAATGAATACTTGGAGGAAGTCGAGGAagtcgcacgacagaaagaaattgaccgcgctaaggcccgtaaagaacgtcacgatgggcgcttagaggcacgcagattcaaacgtcgcaaccgcaaggctcgtaaagcacacaaagctaagcgcgaaagaatgcttgctgcgttgcgatgggctgaacgtcaagaacggcgtaaccatgcggtagctgtagatgtactaggaagaaccaataacgctatgctctgggtcaacatgttc                                                         <  1:457266‑M1/301‑239 (MQ=255)`  
                                                                                                                                                                                                                                                  `cGATTGACCTCTGGGAAGACGCAGAAGACTTGCTCAATGAATACTTGGAGGAAGGCGAGGAagtcgcacgacagaaagaaattgaccgcgctaaggccagtaaagaacgtcacgaggggcgcttataggcacgcagattcaaacgtcgcaaccacaaggcacgtaaagcacacaaagctaagcgcgaaagaatgcttgctgcgtggcgatgggctgaacgtcaagaacggcgtaaccatgaggtagctgtagatgtactaggaagaacaaataacgctatgctctgggtcaacatgttctc                                                       <  1:684997‑M1/301‑241 (MQ=255)`  
                                                                                                                                                                                                                                                            `cttggaagACGCAGAAGACTTGCTCAATGAATACTTGGAGGAAGTCGAGGAagtcgcacgacagaaagaaattgacagcgctaaggcacgtaaagaacgtcacgaggggcgcttagaggcacgcagattcaaacgtcgcaaccgcaaggcacgtaaagcacacaaagctaagcgcgaaagaatgcttgctgcgtggcgatgggctgaacgtcaagaacggcgtaaccatgaggtagctgtagatgtactaggaagaaccaataacgctatgctctgggtcaacatgttctcctgtctcttt                                             >  1:420189‑M1/4‑51 (MQ=255)`  
                                                                                                                                                                                                                                                                 `gagatgtttattagggacaaaatgaatacttggagggagtcgAGGAagtcgcacgacagaaaggaattgaccgcgcttaggcccgtaaagaacgtcacggggggcgcttagaggcacgcagattcaaacgtcgcaaccgcaaggcacggaaagcccacaaagctaagcgcggaagaatgcttgctgcgtggcgattggctgaacgtccagaacggcgtaaccatggggtagctgttgatggtctaggaagaaccaataacgctatgctctgggtcaacatgttctctggggactttaaggc                                        <  1:107061‑M1/281‑256 (MQ=255)`  
                                                                                                                                                                                                                                                                                     `aaagagacagatgaataatgggaggaagtcgcacgaaagaaagaaattgaccgcgcgaaggccagtaaagaacgtcacgaggggcgcttagaggcacgcagattcaaacgtagcaaccgcaaggcacgtaaagcacacaaagctaagcgcgaaagaatgcttgctgcgtggcgatgggctgaacgtcaagaacggcgtaaccatgaggtagctgtagatgtactaggaagaaccaataacgctatgctctgggtcaacatgttctctggggactttaaggcgcttgaggaacgaatcgcgc                    <  1:756155‑M1/280‑276 (MQ=255)`  
                                                                                                                                                                                                                                                                                     `aaTGAATACTTGGAGGAAGTCGAGGAagtcgcacgacagaaagaaattgaccgcgctaaggcccgtaaagaacgtcacgaggggcgcttagaggcacgcagattcaaacgtcgcaaccgcaaggcacgtaaagcacacaaagctaagcgcgaaagaatgcttgctgcgtggcgatgggctgaacgtcaagaacggcgtaaccatgaggtagctgtagatgtactaggaagaaccaataacgctatgctctgggtcaacatgttctctggggactttaaggcgcttgatgaacgaatcgcgc                    <  1:100399‑M1/301‑276 (MQ=255)`  
                                                                                                                                                                                                                                                                                             `cTTGGAGGAAGTCGAGGAagtcgcacgacagaaagaaattgaccgcgctaaggcccgtaaagaacgtcacgaggggcgcttagaggcacgcagattcaaacgtcgcaaccgcaaggcacgtaaagcacacaaagctaagcgcgaaagaatgcttgctgcgtggcgatgggctgaacgtcaagaacggcgtaaccatgaggtagctgtagatgtactaggaagaaccaaaaacgctatgctctgggtcaacatgttctctggggactttaaggcccggcccgtataaacaactccgagccca            >  1:784985‑M1/1‑18 (MQ=255)`  
                                                                                                                                                                                                                                                                                               `tgtaggaagtcgaggaagtcgcacgaaagaaagaaattgaccgcgctaaggcccgtaaagaacgtcacgaggggcgcttagaggcacgcagattcaaacgtcgcaaccgcaaggcacgtaaagcacacaaagctaagcgcgaaagaatgcttgctgcgtggcgatgggctgaacgtcaagaacggcgtaaccatgaggtagctgtagatgtactaggaagaaccaataacgctatgctctgggtcaacatgttctctggggactttaaggcgcttgaggaacgaatcgcgctgcactggcg          <  1:121331‑M1/298‑286 (MQ=255)`  
                                                                                                                                                                                                                                                                                                     `acttggaggaagtcgcacgacagaaagaaattgaccgcgctaaggcccgtaaagaacgtcacgaggggcgcttagaggcacgcagattcaaacgtcgcaaccgcaaggcacgtaaagcacacaaagctaagcgcgaaagaatgcttgctgcgtggcgatgggctgaacgtcaagaacggcgtaaccattaggtagctgtagatgtactaggaagaaccaataacgctatgctctgggtcaacatgttctctggggactttaaggcgcttgaggaacgaatcgcgctgcactggcgtaatgc    <  1:549119‑M1/296‑292 (MQ=255)`  
                                                                                                                                                                                                                                                                                                     `aagtcgaggaaggcgaacgacagaaagaaattgaccgcgctaaggcccgtaaagaacgtcacgaggggcgcttagaggcacgcagattcaaacgtcgcaaccgcaaggcacgtaaagcacacaaagctaagcgcgaaagaatgcttgctgcgtggcgatgggctgaacgtcaagaacggcgtaaccatgaggtagctgtagatgtactaggaagaaccaataacgctatgctctgggtcaacatgttctctggggactttaaggcgcttgaggaacgaatcgcgctgcactggcgtaatgc    <  1:410155‑M1/301‑292 (MQ=255)`  
                                                                                                                                                                                                                                                                                                     `aaggcgaggaagtcgaacgacagaaagaaattgaccgcgctaaggcccgtaaagaacgtcacgagtggcgcttagaggcacgcagattcaaacgtcgcaaccgcaaggcaagtaaagcacacaaagctaagcgcgaaagaatgcttgctgcgtggcgatgggctgaacgtcaagaacggcgtaaccatgaggtagctgtagatgtactaggaagaaccaataacgctatgctctgggtcaacatgttctctggggactttaaggcgcttgaggaacgaatcgcgctgcactggcataatgc    <  1:781594‑M1/301‑292 (MQ=255)`  
                                                                                                                                                                                                                                                                                                       `gtcgaggaagtcgcacgacagaaagaaattgaccgcgctaaggcccgtaaagaacgtcacgaggggcgcttagaggcacgcagattcaaacgtcgcaaccgcaaggcacgtaaagcacacaaagctaagcgcgaaagaatgcttgctgcgtggcgatgggctgaacgtcaagaacggcgtaaccatgaggtagctgtagatgtactaggaagaaccaataacgctatgctctgggtcaacatgttctctggggactttaaggcgcttgaggaacgaatcgcgctgcactggcgtaatgctg  >  1:353683‑M1/1‑8 (MQ=255)`  

                                                                                                                                                                                                                                                                                                          `|`                                                                                                                                                                                                                                                                                                           
`CGTTTTCGACCACGCTTACGAAATGCTGAAAGAAAACATCCGTTATGATGACATCCGTGACACTGATGACCTGCACGATGCTATTCACATGGCTGCCGATAATGCAGTTCCGCACTACTACGCTGACATCTTTAGCGTAATGGCAAGTGAGGGCATTGACCTTGAGTTCGAAGACTCTGGTCTGATGCCTGACACCAAGGACGTAATCCGCATCCTGCAAGCGCGTATCTATGAGCAATTAACGATTGACCTCTGGGAAGACGCAGAAGACTTGCTCAATGAATACTTGGAGGAAGTCGAGGAGTACGAGGAGGATGAAGAGTAATGTCTACTACCAACGTGCAATACGGTCTGACCGCTCAAACTGTACTTTTCTATAGCGACATGGTGCGCTGTGGCTTTAACTGGTCACTCGCAATGGCACAGCTCAAAGAACTGTACGAAAACAACAAGGCAATAGCTTTAGAATCTGCTGAGTGATAGACTCAAGGTCGCTCCTAGCGAGTGGCCTTTATGATTATCACTTTACTTATGAGGGAGTAATGTATATGCTTACTATCGGTCTACTCACCGCTCTAGGTCTAGCTGTAGGTGCA  >  NC_001604.1/954‑1549`

  

Alignment Legend

Aligned base mismatch/match (shaded by quality score): `ATCG/ATCG` `< 3 ≤` `ATCG/ATCG` `< 10 ≤` `ATCG/ATCG` `< 23 ≤` `ATCG/ATCG` `< 35 ≤` `ATCG/ATCG` `< 38 ≤` `ATCG/ATCG`

Unaligned base: `atcg`    Masked matching base: `atcg`    Alignment gap: `‑`    Deleted base: `‑`

Reads not counted as support for junction

read\_name Not counted due to insufficient overlap past the breakpoint.

read\_name Not counted due to not crossing MOB target site duplication.
